# Single-Shot Object Detection via Feature Enhancement and Channel Attention

**DOI:** 10.3390/s22186857

**Published:** 2022-09-10

**Authors:** Yi Li, Lingna Wang, Zeji Wang

**Affiliations:** College of Mathematics and Computer Science, Zhejiang Normal University, Jinhua 321004, China

**Keywords:** deep learning, object detection, feature fusion, attention

## Abstract

Features play a critical role in computer vision tasks. Deep learning methods have resulted in significant breakthroughs in the field of object detection, but it is still an extremely challenging obstacle when an object is very small. In this work, we propose a feature-enhancement- and channel-attention-guided single-shot detector called the FCSSD with four modules to improve object detection performance. Specifically, inspired by the structure of atrous convolution, we built an efficient feature-extraction module (EFM) in order to explore contextual information along the spatial dimension, and then pyramidal aggregation module (PAM) is presented to explore the semantic features of deep layers, thus reducing the semantic gap between multi-scale features. Furthermore, we construct an effective feature pyramid refinement fusion (FPRF) to refine the multi-scale features and create benefits for richer object knowledge. Finally, an attention-guided module (AGM) is developed to balance the channel weights and optimize the final integrated features on each level; this alleviates the aliasing effects of the FPN with negligible computational costs. The FCSSD exploits richer information of shallow layers and higher layers by using our designed modules, thus accomplishing excellent detection performance for multi-scale object detection and reaching a better tradeoff between accuracy and inference time. Experiments on PASCAL VOC and MS COCO datasets were conducted to evaluate the performance, showing that our FCSSD achieves competitive detection performance compared with existing mainstream object detection methods.

## 1. Introduction

Object detection, as a basic, fundamental, and challenging task in computer vision, aims at the detection various visual instances in the real world. With the rapid development of deep neural networks, object detection has been significantly improved in comparison with traditional methods. As the cornerstone of scene understanding and image learning, object detection builds a solid foundation for dealing with other vision tasks, including object tracking [1], image captions [2], segmentation [3], and so on. The emergence of deep learning [4] has provided models with a strong ability to learn sophisticated and complicated representations, leading to remarkable progress in object detection. Generally speaking, there are two main categories of object detectors with deep learning; the first is that of two-stage detectors, which have a higher accuracy but slower inference speed, such as R-CNN [5], SPPNet [6], Fast R-CNN [7], Faster R-CNN [8], Mask R-CNN [9], and R-FCN [10]. The other category is that of one-stage detectors, such as YOLO v1 [11], v2 [12], and its improved versions [13,14], SSD [15], FSSD [16], DSSD [17], and RetinaNet [18], which run faster but with lower accuracy.

It is notable that the quality of features defines the upper limits of a model’s performance, which has been studied by researchers in recent years. The method of using low-level features combined with high-level features is effective for collecting rich information in order to get better detection performance. The feature pyramid network (FPN) [19] was the initial work of enhancing the feature representation by combining multi-level features by building pyramids of different scales. Specifically, shallow feature maps focus on the detection of larger instances, while deeper feature layers are used for smaller ones. Moreover, the design of lateral connections between the bottom-up and top-down layers was introduced in order to deliver the multi-scale details of various objects. Nevertheless, there remain some serious problems in FPNs: The first is information decay [20] during fusion; FPN-based methods adopt 1x1 convolution layers to cause the channel to have the same numbers between the input and the output, but they lose channel information within these operations. For the purpose of a lower computational cost and memory burden, a common practice is the reduction of the number of channels of the feature map, resulting in serious information loss.The second problem is that of aliasing effects [19] during cross-scale fusion. However, there are semantic and contextual differences among feature maps that are to be integrated together. Therefore, simply using *element-add* or concat fusion will lead to serious aliasing effects. Although some previous works [20,21] have ameliorated these issues to a certain degree, there is still much room for further improvement.

In this work, we propose FCSSD in order to enhance the feature representation and alleviate the aliasing affects that originate from FPN feature fusion in object detection. Firstly, inspired by atrous convolution [22], which is expected to capture a larger convolutional receptive size for more information in image segmentation tasks, we present an efficient feature-extraction module (EFM) for exploring the contextual information of a target in the process of a forward pass. The EFM has negligible parameters, has been proven to be effective in strengthening the representation ability of deep features, and contributes to fast and accurate detectors. As we all know, semantic information is vital for the detection of small objects, so a series of downsampling will cause useful details of instances to be lost, as the receptive field is not able to catch different dependencies in the scene. We provide a pyramidal aggregation module (PAM), which includes four levels of kernels with mixed sizes. The PAM improves the ability to process the input features of multi-scale feature maps for capturing rich semantic knowledge about smaller objects. Traditional FPN design has intrinsically restricted the model performance by using a single information flow because of the single lateral connection. In addition, the utilization of element summation is not an effective way to combine features, as it leads to a lack of refinement of the fused features. It is known that low-level features are beneficial for small object identification and detection; however, there is a long pathway for low-level features to reach deeper ones in order to get enough semantic information, thereby increasing the difficulty of accessing accurate localization details. We designed a feature pyramid refinement fusion (FPRF) for generating the refined features for a multi-scale feature map in order to make the most of the shallower and deeper features. Fusing multi-scale feature maps would degrade the power of the model’s representation due to the inconsistent information flow. Furthermore, it would also bring in loss of detail in the information in the highest pyramid due to its the limited channel numbers. Motivated by these innovations, we designed an attention-guided module (AGM), which is a channel attention aggregation module. The AGM aims to optimize the final integrated layers on each level and alleviates the aliasing effects. To validate the performance of our proposed model, we tested our FCSSD on public datasets—PASCAL VOC 2007 and MS COCO 2017. The main contributions of this work can be summarized as follows:

- For feature enhancement, we propose a lightweight efficient feature-extraction module (EFM) and pyramidal aggregation module (PAM). The EFM applies various dilation rates, batch normalization (BN), and ReLU to more richly explore the contextual information of the CNN. The PAM uses different adaptive average pooling sizes to exploit richer semantic information from the deep layers, and upsampling is embraced to keep the same feature size as that of the original input feature map.
- Aiming to make full multi-scale features, we built an effective feature pyramid refinement fusion (FPRF) to calibrate the multi-scale features during the fusion process. The FPRF broadens the ways of the single lateral connections of the traditional FPN and enriches the approaches of multi-scale feature fusion, thus greatly improving the detection performance.
- To alleviate the aliasing effects of the FPN, we introduce an attention-guided module (AGM); an improved channel attention mechanism was developed to ameliorate the problem of fused features, and it is efficient and speeds up the training process.
- By applying the above four improvements, we designed a feature-enhancement and channel-attention-guided single-shot detector (FCSSD). Experiments on the PASCAL VOC2007 and MS COCO2017 datasets showed the effectiveness of our proposed FCSSD and that it can outperform mainstream object detectors.

## 2. Related Work

### 2.1. Deep-Learning-Based Object Detectors

Deep-learning-based object detection can be divided into the categories two-stage detectors and one-stage detectors. R-CNN [5] was first to show that the use of a convolutional neural network (CNN) could enable one to reach unprecedented object detection performance on the PASCAL VOC [23] dataset. R-CNN firstly produces fixe-length features from generated proposals and then uses linear SVMs and a bounding box regressor to classify objects for a precise bounding-box prediction. Although R-CNN represents a breakthrough in object detection, it is a multi-stage pipeline and has a higher computational cost, and it is slow in its running time because each part of the whole network must be trained separately. SPPNet [6] introduced a spatial pyramid pool (SPP) module with the aim of obtaining a fixed length for fully connected layers. In an evaluation, the SPP was shown to be faster than the R-CNN to some extent, but still could not achieve real-time object detection. Fast R-CNN [7] makes the combination of classification and regression, achieving a fast end-to-end training paradigm. Faster R-CNN [8] involved the development of a region proposal network, requiring nearly cost-free region proposals and boosting the performance significantly. Cascade R-CNN [24] was used to study the effect of regression proposals for under different IoU thresholds based on faster R-CNN and achieved better performance in object detection. In addition to these, there are still many other excellent two-stage detectors, such as Mask R-CNN [9], R-FCN [10], and CBNet [25].

One-stage detectors usually apply a unified network to directly accomplish localization and classification with more efficiency but lower accuracy. YOLO [11] frames the detection task as a regression problem and divides the input image into some grids; thus, extracting features from the input image results in the direct prediction of the bounding box and classification from each unit of the grid within a united architecture. For the sake of achieving real-time detection without giving away much accuracy, SSD [15] combines the idea of the RPN in Faster R-CNN, YOLO, and multi-scale convolutional features to achieve fast detection. RetinaNet [18] introduces a new balanced loss function called focal loss to downweight the contributions of an easy sample v.s. a hard example, thus improving the detection performance of one-stage detectors. There are also other one-stage detectors, including the YOLO series [12,13,14], FSSD [16], and DSSD [17].

### 2.2. Enhancement of Feature Representation

Deep learning techniques have a powerful ability to learn rich feature representations with multi-level features directly from raw images. The extraction of effective features is a critical issue for more accurate classification and localization. Earlier object detectors usually directly performed predictions based on the pyramid feature hierarchy extracted from a backbone network [15]. In a pioneering work, the effective extraction of the low-level features of a network was determined to be a key problem in object detection. The feature pyramid network (FPN) [19] was the first to enhance a CNN’s representation by fusing features from different levels and constructing feature pyramids. The FPN proposes a top-down pathway and uses lateral connections to combine multi-level features. The design of the FPN shows that fully utilizing the multi-scale features can promote accuracy in object detection. PFPNet [26] was used to investigate the width of the FPN by means of building feature pyramid blocks to widen the network and further improve the performance. PANet [27] was used to explore an extra bottom-up pathway to improve the low-level information in deep layers. The proposal of TridentNet [28] was the first to use the effects of the receptive field in object detection with different scale sizes, and it was constructed with a parallel multi-branch architecture in which there were three branches for training, but only one of them was used for testing, which ensured that no additional parameters or computations were added during forward inference. EfficientDet [21] used a weighted bi-directional FPN to perform easy and fast feature fusion. RefineDet [29] used an anchor-refined module and object detection module to get better features after refinement and fusion, achieving excellent accuracy and high efficiency.

### 2.3. Attention Mechanisms

Attention mechanisms imitate human cognitive awareness about specific information, amplifying critical details to focus more on the essential aspects of data. In addition, attention mechanisms are able to build long-range dependencies within a model and become the workhorse of many challenging tasks, including image classification [30], semantic and instance segmentation [31], and natural language processing [32]. Attention mechanisms have a wide range of applications in object detection because they help the model to better locate and recognize objects in images, thus further improving detection performance [33,34]. SENet [35] involved a squeeze−and−excitation (SE) block with the aim of collecting global information along with channel-wise relationships and strengthening the representation ability of a CNN with efficient operations. ECANet [36] involved an efficient−channel−attention (ECA) block, a local cross-channel interaction strategy without dimensionality reduction implemented through one-dimensional convolution, which improved the training speed with a lower model complexity. GENet [37] mainly included two operations, Gather and Excite. The former efficiently aggregated feature responses over a large spatial area, and the latter redistributed the combined information to local features in a spatial domain. SKNet [38] involved selectivekernel (SK) convolution, which enabled the network to adjust the kernel size of the convolution operation according to the input, achieving significant performance gains at a small computational cost. CBAM [39] was used to study the effects of model performance in the spatial dimension and channel dimension. For CBAM, two parallel branches with max pooling and average pooling were designed to explore the interrelations of features between channels, and a concatenation operation was employed to gather the final outputs. CABM told the network where to focus and where to pay attention by modeling the spatial and channel dimensions of the features.

Based on the methods listed above, we focused on building an effective method for exploring multi-level feature fusion in the FPN; thus, we propose the FCSSD. The FCSSD consists of four modules—the EFM, PAM, FPRF, and AGM—based on SSD. The details of FCSSD are introduced in the following sections.

## 3. Methodology

**Figure 1:**
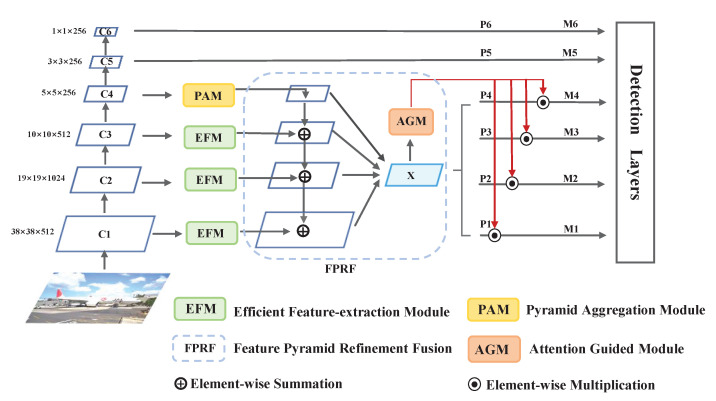
Overall architecture of the proposed FCSSD. Our approach consists of four components: a standard SSD network; feature-enhancement components: the efficient feature-extraction module (EFM) and pyramidal aggregation module (PAM); the feature pyramid refinement fusion (FPRF) process *X*; the attention-guided module (AGM). Here, $C_{i}$ denotes the feature map extracted from the CNN backbone, $P_{i}$ denotes the corresponding pyramid levels in the FCSSD, and $M_{i}$ is the final output of every level. An input image is first downsampled and passed through the EFM and PAM to produce features with more semantic and rich global contextual information. The FPRF uses an FPN-style design to refine the previous features for fusion. The AGM adopts a channel attention scheme to extract channel-balanced weights, and it reduces the aliasing effects of the fused features.

In this section, each part of the proposed FCSSD, which is shown in Figure 1, is demonstrated in detail. First, we introduce the specific design of our network in Section 3.1. Then, the efficient feature-extraction module (EFM) is presented in Section 3.2. Next, the pyramidal aggregation module (PAM) is shown in Section 3.3. Section 3.4 describes the feature pyramid refinement fusion (FPRF). Finally, in Section 3.5, the attention-guided module (AGM) is explained.

### 3.1. FCSSD Architecture

In this work, we adopt a one-stage SSD for object detection as our baseline due to its good tradeoff between high speed and detection accuracy. The SSD predicts layers to match the output, including both the bounding box regression and classification. Its object detection results are from six final feature maps; the adjusted VGG-16 is used as the backbone, and each feature map predicts different scales of an individual object. NMS (non-maximum suppression) is usually adopted to filter redundant and overlapping predictions to generate the final detection result.

Figure 1 shows the overall architecture of our proposed method, consisting of four parts: the standard SSD network; feature enhancement fusion component: the efficient feature-extraction module (EFM), pyramidal aggregation module (PAM), feature pyramid refinement fusion (FPRF) process *X*, and attention-guided module (AGM). As we mentioned before, the standard SSD employs VGG-16 as the network backbone. We then regard the six layers of the SSD as the prediction feature maps. The strides are {8,16,32,64,100,300} pixels of multiple feature maps with respect to the input image size of 300×300. For brevity, we refer to these as C1, C2, C3, C4, C5, and C6. The EFM exploits rich contextual information from receptive fields with different sizes, and the PAM collects prior global-scene-level semantic knowledge from the CNN. An effective FPRF is applied to the multi-scale features to generate a refined map *X*. The AGM is a channel-attention-guided module that reduces the aliasing effects for fused feature maps and accelerates the training speed. We will discuss the above modules and fusion process in the following subsections.

### 3.2. Efficient Feature-Extraction Module

**Figure 2:**
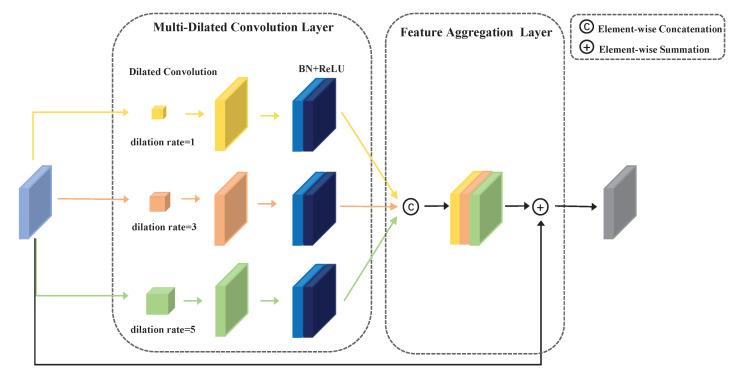
The architecture of the efficient feature-extraction module (EFM). The multi-dilated convolution layer has three different dilation rates: 1, 3, and 5. The feature aggregation layer contains two operations: element-wise concatenation and element-wise summation.

As we know, object detection requires contextual information, especially for small objects [40]. The efficient feature-extraction module (EFM) was inspired by the mode in which humans distinguish objects by relying on different sizes, colors, backgrounds, and shapes. For example, it is difficult for a human to distinguish a bird very high in the sky, but it is easy to recognize when the sky is taken into consideration as the contextual information. Therefore, we believe that contextual knowledge is helpful for object detection. The EFM takes advantage of dilated convolution [22] to exploit larger receptive fields on the feature map, thus acquiring a considerable contextual information about the detected objects. To improve the accuracy of the multi-scale object detection, we apply dilated convolution with three kinds of dilation rates to obtain the contextual information from the receptive fields. As Figure 2 shows, the EFM has a multi-branch convolutional design, and it has two main components: a multi-dilated convolution layer and a feature aggregation layer.

For the input features $R^{C\times H\times W}$, the multi-dilated convolution layer has parallel branches consisting of dilated convolution, a BN layer, and the ReLU activation function. Three different dilation rates are used, but with the same kernel size. Specifically, the kernel of the dilated convolution is conv3×3, and the dilation rates are 1, 3, and 5 for the different branches. We also use the same padding to keep the input and output feature maps at the same size. We adopt a scale ratio *r* to reduce the channels of the feature map for efficiency. This process can be expressed as:

(1) $$F_{out}=Conv_{d(1,3,5)}R^{\frac{C}{r}\times H\times W}$$

where $R^{\frac{C}{r}\times H\times W}$ is the input feature map with a reduced channel ratio; *C*, *H*, and *W* refer to the channel, height, and width of the feature map; *r* equals 8 as the hyper-parameter; $Conv_{d(1,3,5)}$ indicates conv3×3 with three different dilation rates of 1, 3, and 5; $F_{out}$ refers to the output feature map.

The feature aggregation layer is used to fuse contextual information from different parallel branches and make the most of the diverse feature details. Element concatenation and summation are adopted to produce the multi-branch feature representation. It is notable that stacks of conv1×1 and conv3×3 are used to reduce the channels of the concatenation feature map. As residual learning, the input feature map is added to that. The whole process can be expressed as:

(2) $$F_{out}=Conv\left(Concat\left[Conv_{d(1,3,5)}R^{\frac{C}{r}\times H\times W}\right]+R^{C\times H\times W}\right)$$

### 3.3. Pyramidal Aggregation Module

Deep convolutional neural network (DCNN) methods have strengthened object detection performance by a great margin, but still face tough challenges when considering different scenes, object sizes, and backgrounds. Semantic relationships are universal and important for object detection, especially when the target is very small [21,27]. Prior works proved that not collecting the necessary semantic information increases the possibility of misclassification and mislocalization. Overlooking the global scene information may result in failure to consider an object’s details, such as its pattern, texture, and shape. Therefore, one should focus on separate subregions that contain inconspicuous information of interest. In order to learn more suitable prior global-scene-level knowledge for the CNN, we built a pyramidal aggregation module (PAM). This was aimed at the aggregation of global semantic information along with subregions, as well as at the reduction of the training loss between different subregions.

**Figure 3:**
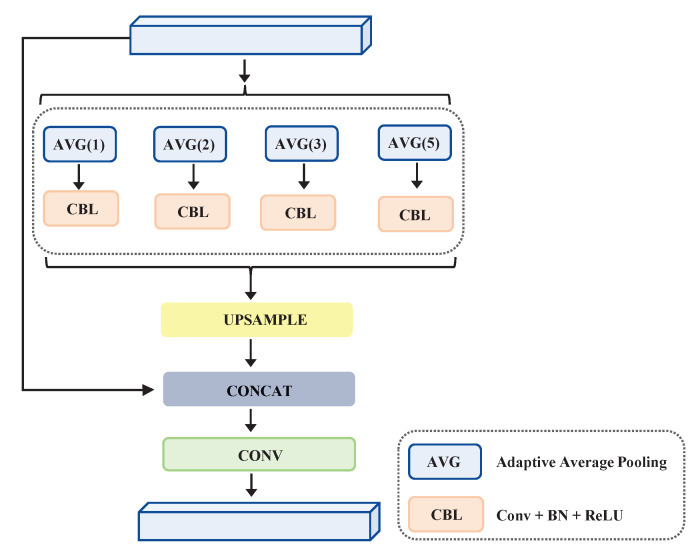
Pyramidal aggregation module (PAM).

Global average pooling is commonly used in image classification tasks by enforcing correspondences between feature maps and categories. The PAM fuses features with four different pyramid scales. As shown in Figure 3, a stack of pyramid levels with four different AVG sizes is used to separate the input features and form pooled representations for locations; it is followed by CONV, BN, and ReLU. The input features first go through the average pooling pyramid to generate the aggregation information. Next, all of the aggregated features are upsampled through bilinear interpolation, followed by the concatenation of the original input feature to produce rich semantic information. Finally, a series of conv operations are used to balance the channel number. The whole process can be formulated as:

(3) $$F_{out}=Conv\left(Concat\left[Upsample\left(Avg_{k=1,2,3,5}\left(F_{in}\right)\right),F_{in}\right]\right)$$

where $F_{in}$ and $F_{out}$ refer to the input and output feature maps, respectively. Conv is Conv3×3 operations, and Concat is the concatenation for aggregating all of the feature maps. Upsamle indicates an upsampling operation for the generated output. Avg is adaptive average pooling with different sizes of 1, 2, 3, and 5.

### 3.4. Feature Pyramid Refinement Fusion

**Figure 4:**
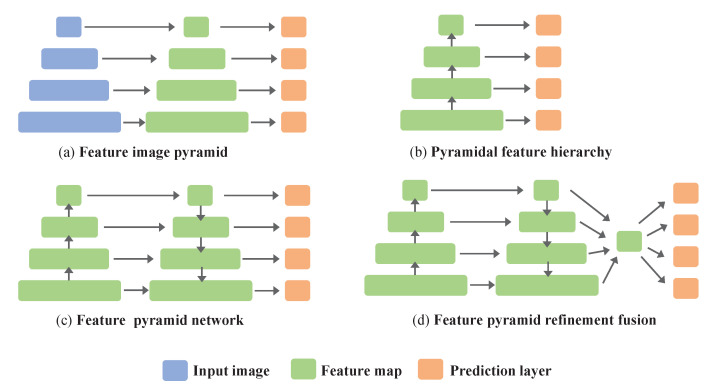
(**a**) Using an image pyramid to build a feature pyramid, which is an inefficient method [41]. (**b**) Reusing the pyramidal feature hierarchy computed by a ConvNet as if it were a featurized image pyramid, as with a single-shot multi-box detector (SSD) [15]. (**c**) Bottom-up and top-down pathway feature fusion, as in an FPN [19]. (**d**) Our proposed feature pyramid refinement fusion.

Traditional FPN models are inefficient in exploring stacks of feature maps that contain a wide range of scales, giving rise to inferior detection performance. The SSD [15] introduced the design of a pyramidal feature hierarchy for detecting multi-scale objects, as shown in Figure 4b. However, it failed to exploit the relationships of the information between shallower layers and deeper layers and just used a single-stage feature map while neglecting the complementary effects of all layers, which made it hard to achieve good performance for small object detection. To alleviate this problem, an intuitive notion is that of acquiring multi-scale features, such as with an FPN [19], as shown in Figure 4c. However, the information flow in the FPN becomes another critical problem, as the lateral connections and top-down pathways greatly limit the upper bound of feature utilization. To improve the model’s efficiency and enrich the single lateral connections of the traditional FPN, we propose feature pyramid refinement fusion. Different input resolutions contribute unequally to the final detection results because they carry different characteristic features of the object. If we gather them together into a refined feature, then they will contain different information at different scales. Finally, we just apply the refined feature map to previous features through multiplication to get the final output for detection. This process can be formulated as:

(4) $$\begin{array}{cc} P_{4}^{out} & =Conv(C_{4}^{in})\\ P_{3}^{out} & =Conv(C_{3}^{in}+Upsample\left(P_{4}^{out}\right))\\ P_{2}^{out} & =Conv(C_{2}^{in}+Upsample\left(P_{3}^{out}\right))\\ P_{1}^{out} & =Conv(C_{1}^{in}+Upsample\left(P_{2}^{out}\right)) \end{array}$$

(5) $$\begin{array}{cc} R_{4} & =Resize(P_{4}^{out},S)\\ R_{3} & =Resize(P_{3}^{out},S)\\ R_{2} & =AMP(P_{2}^{out},S)\\ R_{1} & =AMP(P_{1}^{out},S)\\ X & =\frac{\left(R_{1}+R_{2}+R_{3}+R_{4}\right)}{4} \end{array}$$

(6) $$\begin{array}{cc} M_{i} & =R_{i}\times X,\;i\in \left\{1,2,3,4\right\}\\ M_{j} & =P_{j},\;j\in \left\{5,6\right\} \end{array}$$

where AMP refers to adaptive max pooling, *S* is the scale size of feature map $C_{2}$, *X* represents the refined features, and *M* refers to the final feature maps for prediction.

### 3.5. Attention-Guided Module

**Figure 5:**
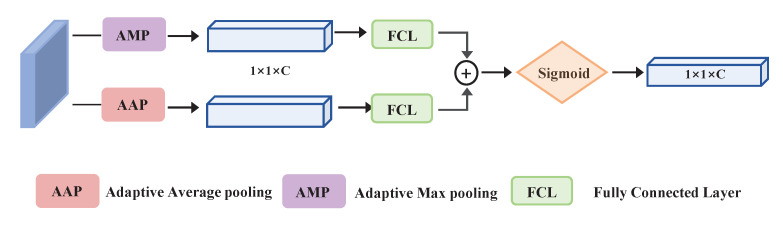
Structure of the attention-guided module (AGM).

The multi-scale features are used to improve small object detection to some extent. Nevertheless, serious semantic differences exist among features of multiple shapes, especially in the fusion process. Miscellaneous integrated information may lead to aliasing effects [19,42], causing the mistakes in localization and classification. In the original FPN, a series of 1×1 and 3×3 convolutions is usually followed by each fused feature map to reduce these effects. Features generated from the VGG-16 backbone contain more serious aliasing effects after the EFM and PAM. In order to mitigate the negative influences of the aliasing effects, it is appropriate to employ an attention mechanism on the fused feature maps. However, just applying attention modules on integrated features would bring in a vast computational burden because the SSD takes on six feature maps. We hope that the attention mechanism will not only alleviate this aliasing effect, but also that it will require fewer parameters. Inspired by the channel attention design of CBAM [39], we developed an attention-guided module (AGM), as illustrated in Figure 5, to solve the inconsistency between layers and mitigate the aliasing effects between multi-scale feature maps. Two different types of spatial contextual information are generated by employing the two main pooling paradigms (AMP and AAP). These two kinds of information descriptors independently head to the FC layers. Element-wise summation and a sigmoid activation function are used to get the final output. The whole process can be expressed as:

(7) $$\begin{array}{cc} AFG & =\sigma (FC\left(GAP\left(F_{in}\right)\right)+FC\left(GMP\left(F_{in}\right)\right))\\ M_{i} & =AFG\left(X\right)\times P_{i} \end{array}$$

where *i* refers to the index of the pyramid levels in the backbone, $P_{i}$ refers to fused features, $M_{i}$ refers to the final outputs, $F_{in}$ and AFG are the input and output, respectively, σ is a sigmoid activation function, FC is the fully connected layer, and GAP and GMP refer to global average pooling and global max pooling, respectively.

## 4. Results

### 4.1. Dataset and Experimental Details

We used the PASCAL VOC 2007 [23] and MS COCO 2017 [40] datasets to validate the model proposed in this work. Training was performed on the VOC 2007 trainval data with 20 classes, which were in combination with 5k images and the VOC 2012 trainval dataset. The evaluation was performed on the VOC 2007 test set with 5k images. Here, we used the mAP as the evaluation criterion for this dataset. The input was 300×300, the batch size was set to 16 with a total of 250 epochs during training on a single 2080Ti GPU, and the learning rate was $4\times 10^{-3}$ at the beginning. The warming-up training strategy was used to adjust the learning rate, which gradually increased the learning rate from $6\times 10^{-1}$ to $4\times 10^{-3}$ in the first five epochs. It dropped the original point and was divided by 10 at 150, 200, and 230 epochs. The weight decay was set to $1\times 10^{-5}$, and the momentum was set to $9\times 10^{-1}$. For the input size of 512×512, the total of the training epochs was up to 200, the batch size was decreased to 8, and the other settings were kept unchanged.

**Figure 6:**
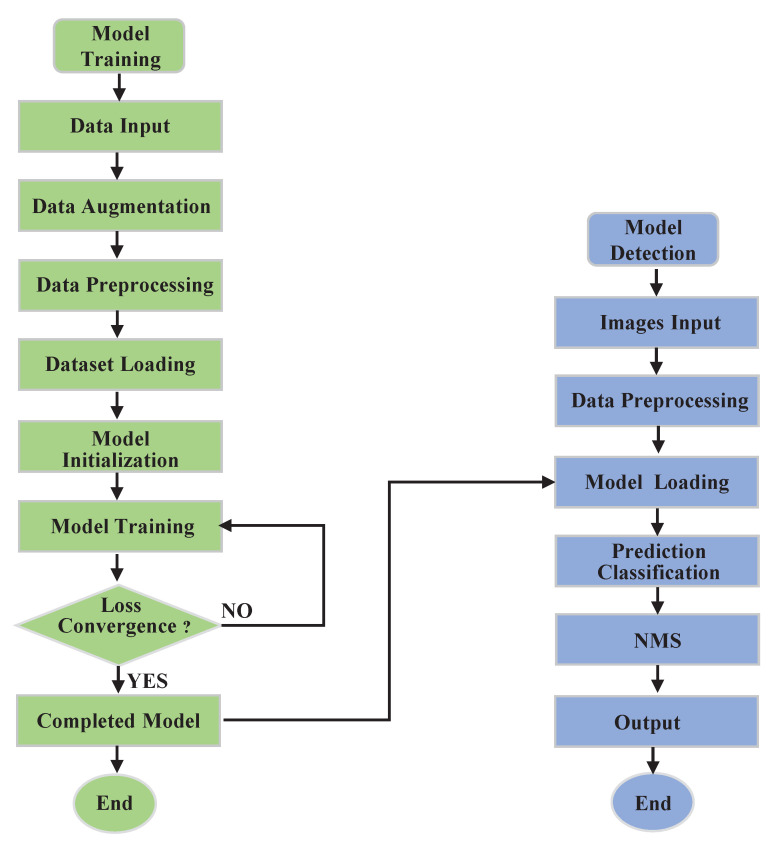
Flow diagram of the training and detection process [43].

Our FCSSD was trained based on the MS COCO 2017 dataset, which contains 80 classes, 115k images for training (train2017), 5k images for validation (val2017), and 20k images for testing (testdev). We set the total number of training epochs to 150, and also adopted the warming-up technique to increasingly raise the learning rate from $6\times 10^{-1}$ to $4\times 10^{-3}$ in the first five epochs and then decreasing it after 60 and 100 epochs by a factor of 10, finishing up at 140 epochs. With these experimental settings, we used a batch size of 8, and the weight decay, momentum, and other settings were the same as those with the PASCAL VOC training strategy. Table 1 shows detailed information on the two datasets. Figure 6 provides a detailed workflow of the training and detection process for the improved model used in this paper.

### 4.2. Evaluation Metric

To evaluate the model’s performance in object detection, the precision, recall, and mean average precision (mAP) were used. They can be expressed as:

(8) $$Precision=\frac{TP}{TP+FP}$$

(9) $$Recall=\frac{TP}{TP+FN}$$

where TP indicates true positives, FN indicates false negatives, and FP indicates false positives.

The intersection over union (IoU) was used to set the thresholds of the ground-truth box and the prediction box to determine the truth.

(10) $$IoU=\frac{area(B_{pred}\cap B_{gt})}{area(B_{pred}\cup B_{gt})}$$

where $B_{pred}$ indicates the predicted bounding box, and $B_{gt}$ is the ground-truth bounding box. The IoU threshold in PASCAL VOC was set to 0.5, and the mAP of the MS COCO dataset ranged from 0.5 to 0.9 with a step size of 0.05.

(11) $$mAP=\frac{mAP_{0.50}+mAP_{0.55}+mAP_{0.60}+...+mAP_{0.90}+mAP_{0.95}}{10}$$

In addition to the basic evaluation of the mAP, the average precision (AP) was also included. AP, $AP_{50}$, $AP_{75}$, $AP_{small}$, $AP_{medium}$, and $AP_{large}$ depended on different thresholds from those of the criteria for MS COCO.

### 4.3. Experimental Analysis

#### 4.3.1. PASCAL VOC 2007

We compared our method with the mainstream one-stage and two-stage object detectors, as shown in Table 2. Obviously, in order to achieve better performance, most existing two-stage detectors usually have a larger input size (typically 1000×800). CoupleNet [44] achieved an mAP of 82.7, which was 1.2 higher than the mAP of our method with a 300×300 input. For the one-stage methods, we used two input variants for a fair comparison: 300×300 and 512×512 scales. The baseline SSD [15] achieved detection with an mAP of 77.2 with a 300×300 input. Our approach provided a considerable increase of 4.3% in terms of mAP in comparison with the baseline SSD. Our FCSSD surpassed the DSSD [17], which used a strong feature extraction backbone, ResNet-101, by a large margin of 2.9%. In comparison with the complicated FPN-style methods, such as PFPNet [26] and RefineDet [29], our method achieved a slightly higher mAP than they did, and this highlighted the effectiveness of our proposed FCSSD. With the input size of 512×512, RefineDet [29], RFBNet [45], and PFPNet [26] achieved accuracies of 81.8, 82.2, and 82.3, respectively. With the same input size and backbone, our method outperformed them with an accuracy of 83.2 mAP. Table 2 shows the results of our FCSSD versus those of mainstream object detectors on this dataset. The FPS evaluation metric was used to examine the model’s inference speed, and our FCSSD also achieved a better tradeoff between accuracy and speed.

#### 4.3.2. MS COCO

Table 3 shows the results on the MS COCO dataset. For a 300×300 input, the baseline SSD [15] achieved a detection performance score of 25.1. With the same feature extraction backbone, our method achieved a notable refinement of 13.1% for the overall detection score in comparison with the baseline SSD. For large objects $\left(AP_{l}\right)$, the baseline SSD attained a pure performance of 41.4 AP. However, it seriously deteriorated to 6.6 AP for small objects $\left(AP_{s}\right)$, as it showed inferior quality in small object detection. Significantly, our FCSSD achieved a detection performance of 15.3 AP for this criterion, which was over double compared with the SSD baseline. Likewise, we also accomplished a great margin of improvement in detection performance with medium objects $\left(AP_{m}\right)$. Among the existing single-stage detectors, RFBNet [45] and RefineDet320+ [29] provided scores for overall detection of 30.3 and 35.2, respectively. With the same VGG-16 backbone, our detector achieved results superior to those obtained with both methods.

For the 512×512 input size, the baseline SSD achieved an overall detection score of 28.8. Our method provided a significant gain of 12.7% in terms of AP with the same VGG backbone. Among the existing methods, EfficientDet-D1 [21] and YOLOv4 [14] provided detection AP scores of 39.6 and 41.2, respectively, due to their efficient framework design. Compared with the anchor-free-style method CornerNet [54], our method showed a slightly better performance in terms of AP and outperformed it in terms of inference time (65 versus 227 ms), which can be expensive in terms of costs when processing an image. The low quality of feature representations of small objects was due to their limited size and the general feature extraction, which is still a tough and challenging problem in both types of detectors. The experiments demonstrated that the feature representation power could be strengthened through our FCSSD network design. In the MS COCO dataset, more than 70% of the images are composed of objects, the size of which is usually less than 32×32 pixels in one image. In addition, MS COCO contains more detailed information on the objects than the images in PASCAL VOC do, and this can further enhance the learning ability of our method.

The two-stage detectors were able to achieve superior accuracy, but with a high computational cost. They usually require a considerably larger input resolution and need more than 100 ms to process an image during the inference time. For example, Mask-RCNN [9] achieved an AP of 39.8, but needed 210 ms to infer an image. Our method provided competitive accuracy with a high efficiency and achieved excellent performance, with a detection AP score of 41.5 and an inference time of 65 ms. This shows that our FCSSD not only achieves better performance, but also operates at high efficiency.

#### 4.3.3. Ablation Study

To verify the effectiveness of the proposed modules in our FCSSD, we conducted various ablation experiments on the PASCAL VOC 2007 test set and MS COCO minival. To be specific, we used VGG-16 as the backbone and an input size of 300×300 in all experiments. As shown in Table 4, when we used only the EFM, the mAP was increased by 1.4% in comparison with that of the baseline SSD (77.2%) [15]. Based on the EFM, we found that the mAP was further increased from 78.6% to 79.5% with the embedding of the PAM. The FPRF refined multi-scale features from the shallow layers and deep layers and showed a more effective feature fusion method, which boosted the performance by 2% in comparison with that of the original SSD. Since the AGM plays an important part in balancing the channel weights of features and reduces the aliasing effects caused by the FPN design, it boosted the performance of the model by 1% based on the FPRF, from 79.2% to 80.2%. Finally, the FCSSD reached an mAP of 81.5% on the PASCAL VOC2007 test set with the image size of 300×300. As shown in Table 5, the AP of the baseline SSD gradually increased as we added the EFM and PAM to the model. When the FPRF was applied to the model, the AP underwent a large increase, showing that multi-scale feature pyramids are beneficial for object detection and provide richer semantic and spatial information. The AGM was intended to reduce the redundancy and balance the channel weights, and it further increased the AP to 38.2. These experimental results validate the effectiveness of our modules; our FCSSD performs accurately and efficiently.

**Figure 7:**
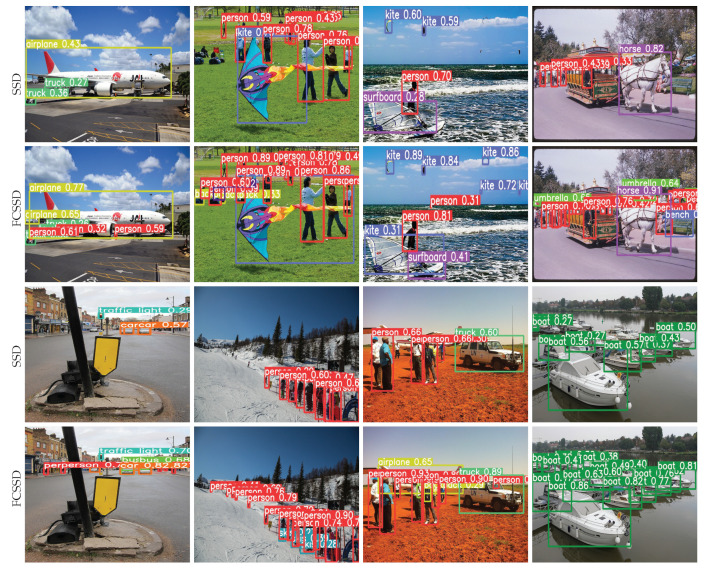
Qualitative results of the SSD in comparison with those of the FCSSD on the PASCAL VOC and MS COCO datasets.

We show some qualitative results of the SSD [15] in comparison with those of our FCSSD on the PASCAL VOC 2007 test set and MS COCO minival in Figure 7. The figure compares images containing objects of various sizes. When the FCSSD was applied to the test images, even small objects were successfully detected due to the effectiveness our model design.

## 5. Conclusions

In this work, we proposed a feature-enhancement- and channel-attention-guided single-shot detector for object detection called the FCSSD. Our method brings in four effective improvements: an efficient feature-extraction module (EFM), pyramidal aggregation module (PAM), feature pyramid refinement fusion (FPRF), and attention-guided module (AGM). The EFM is designed to exploit multi-scale contextual information in the shallow layers, while the PAM is used to explore richer semantic knowledge in the deep layers. The FPFR introduces an effective means of feature fusion for a PFN by compressing multi-scale features into a refined feature. The AGM balances the channel weights and deals with the aliasing effects of the fused features in the FPN, thus accelerating the training process and further improving the performance. Experiments on public datasets proved that our approach achieved competitive results in comparison with those of mainstream one-stage and two-stage methods, achieving better a tradeoff between accuracy and speed. Since one-stage object detectors achieve a faster speed and better performance, further research is needed to move on to lightweight models for real applications that could easily be embedded into mobile devices. 

## Figures and Tables

**Table 1:** Dataset statistics of PASCAL VOC and MS COCO.

Name	Train Images	Validation Images	Test Images	Category
VOC 2007 [23]	2501	2510	4952	20
MS COCO 2017 [40]	11287	5000	40670	80

**Table 2:** Comparison of our method with existing detectors on the PASCAL VOC 2007 test set (with IoU = 0.5); 07 + 12: 07 trainval + 12 trainval.

Method	Training Data	Backbone	Input size	mAP	FPS
**Two-stage detectors:**					
Faster R-CNN [8]	07 + 12	VGG-16	∼1000×600	73.2	7
Faster R-CNN [8]	07 + 12	ResNet-101	∼1000×600	76.4	5
R-FCN [10]	07 + 12	ResNet-101	∼1000×600	80.5	9
CoupleNet [44]	07 + 12	VGG-16	∼1000×600	82.7	8
ION [46]	07 + 12	VGG-16	∼1000×600	79.2	1.3
**One-stage detectors:**					
SSD [15]	07 + 12	VGG-16	300×300	77.2	46
RON320++ [47]	07 + 12	VGG-16	320×320	76.6	20
DSSD [17]	07 + 12	ResNet-101	321×321	78.6	10
R-SSD [48]	07 + 12	VGG-16	300×300	78.5	35
YOLOv2 [12]	07 + 12	DarkNet-19	544×544	78.6	40
StrairNet [49]	07 + 12	VGG-16	300×300	78.8	30
DES [50]	07 + 12	VGG-16	300×300	79.7	76
PFPNet [26]	07 + 12	VGG-16	300×300	80.0	40
RFBNet [45]	07 + 12	VGG-16	300×300	80.5	83
RefineDet [29]	07 + 12	VGG-16	320×320	79.7	76
**FCSSD (Ours)**	07 + 12	VGG-16	300×300	**81.5**	55
SSD [15]	07 + 12	VGG-16	512×512	77.2	46
FSSD [16]	07 + 12	VGG-16	512×512	80.9	35.7
DES [50]	07 + 12	VGG-16	300×300	81.7	31
RefineDet [29]	07 + 12	VGG-16	512×512	81.8	21
RFBNet [45]	07 + 12	VGG-16	512×512	82.2	38
PFPNet [26]	07 + 12	VGG-16	512×512	82.3	26
**FCSSD (Ours)**	07 + 12	VGG-16	512×512	**83.2**	35

**Table 3:** The results of the mainstream one-stage and two-stage detectors on the MS COCO testdev.

Method	Backbone	Input Size	Time (ms)	AP	${\mathrm{AP}}_{50}$	${\mathrm{AP}}_{75}$	${\mathrm{AP}}_{s}$	${\mathrm{AP}}_{m}$	${\mathrm{AP}}_{l}$
**Two-stage detectors:**									
Faster R-CNN [8]	VGG-16	1000×600	147	24.2	45.3	23.5	7.7	26.4	37.1
Faster FPN [19]	ResNet-101-FPN	1000×600	240	36.2	59.1	39.0	18.2	39.0	48.2
CoupleNet [44]	ResNet-101	1000×600	121	34.4	54.8	37.2	13.4	38.1	50.8
Mask R-CNN [9]	ResNetXt-101-FPN	1280×800	210	39.8	62.3	43.4	22.1	43.2	51.2
Cascade R-CNN [24]	ResNet-101-FPN	1280×800	141	42.8	62.1	46.3	23.7	45.5	55.2
**One-stage detectors:**									
SSD [15]	VGG-16	300×300	20	25.1	43.1	25.8	6.6	25.9	41.4
DSSD [17]	ResNet-101	321×321	-	28.0	46.1	29.2	7.4	28.1	47.6
RetinaNet [18]	ResNet-101	500×832	90	34.4	53.1	36.8	14.7	38.5	49.1
DES [50]	VGG-16	300×300	-	28.3	47.3	29.4	8.5	29.9	45.2
RFBNet [45]	VGG-16	300×300	15	30.3	49.3	31.8	11.8	31.9	45.9
EFIPNet [51]	VGG-16	300×300	14	30.0	48.8	31.7	10.9	32.8	46.3
RefineDet320+ [29]	VGG-16	320×320	-	35.2	56.1	37.7	19.5	37.2	47.0
M2det [52]	VGG-16	320×320	-	38.9	59.1	42.4	24.4	41.5	47.6
**FCSSD(ours)**	VGG-16	300×300	35	**38.2**	**59.4**	41.5	15.3	40.3	**58.6**
YOLOv2 [12]	DarkNet	544×544	25	21.6	44.0	19.2	5.0	22.4	35.5
YOLOv3 [13]	DarkNet-53	416×416	35	31.0	55.3	32.3	15.3	33.2	42.8
SSD [15]	VGG-16	512×512	28	28.8	48.5	30.3	10.9	31.8	43.5
DSSD [17]	ResNet-101	513×513	156	33.2	53.3	35.2	13.0	35.4	51.1
RefineDet [29]	VGG-16	512×512	45	33.0	54.5	35.5	16.3	36.3	44.3
RefineDet [29]	ResNet-101	512×512	-	36.4	57.5	39.5	16.6	39.9	51.4
RFBNet-E [45]	VGG-16	512×512	30	34.4	55.7	36.4	17.6	37.0	47.6
TripleNet [53]	ResNet-101	512×512	-	37.4	59.3	39.6	18.5	39.0	52.7
EfficientDet-D1 [21]	EfficientNet-B1	640×640	50	39.6	58.6	42.3	17.9	44.3	56.0
CornerNet [54]	Hourglass-104	511×511	227	40.5	56.5	43.1	19.4	42.7	53.9
YOLOv4 [14]	CSPDarkNet53	416×416	38	41.2	62.8	44.3	20.4	44.4	56.0
M2det [52]	VGG-16	512×512	-	42.9	62.5	47.2	28.0	47.4	52.8
Scaled-YOLOv4 [55]	CD53	608×608	-	45.5	64.1	49.5	27.0	49.0	56.7
YOLOX-M [56]	Modified CSP v5	640×640	-	46.4	65.4	50.6	26.3	51.0	59.9
**FCSSD(ours)**	VGG-16	512×512	65	**41.5**	**63.1**	46.9	21.8	46.2	**56.5**

**Table 4:** Ablation studies of the FCSSD on the PASCAL VOC 2007 test set with an image size of 300×300.

Method	EFM	PAM	FPRF	AGM	mAP
Baseline SSD					77.2
(a)	✓				78.6
(b)		✓			78.4
(c)			✓		79.2
(d)	✓	✓			79.5
(e)			✓	✓	80.2
**FCSSD (ours)**	✓	✓	✓	✓	81.5

**Table 5:** Ablation studies of the FCSSD on the MS COCO minival with an image size of 300×300.

Scheme	Methods	AP	${\mathrm{AP}}_{50}$	${\mathrm{AP}}_{75}$	${\mathrm{AP}}_{s}$	${\mathrm{AP}}_{m}$	${\mathrm{AP}}_{l}$
A	Baseline SSD	25.1	43.1	25.8	6.6	25.9	41.4
B	A + EFM	27.2	45.2	27.2	7.9	27.3	44.6
C	B + PAM	29.3	47.9	30.6	9.1	30.1	45.2
D	C + FPRF	37.5	56.9	38.8	13.5	38.3	54.2
E	D + AGM	38.2	59.4	41.5	15.3	40.3	58.6

## Data Availability

PASCAL VOC: http://host.robots.ox.ac.uk/pascal/VOC/; MS COCO: https://cocodataset.org/ accessed on 9 July 2022.
